# Xiphodynia as an Unusual Cause of Chest Pain: A Case Series

**DOI:** 10.1055/s-0043-1761270

**Published:** 2023-02-07

**Authors:** Anniek van Boekel, Guido Stollenwerck, Ewan D. Ritchie, Sanne Vogels

**Affiliations:** 1 Department of Surgery, Alrijne Hospital, Leiderdorp, The Netherlands; 2 Department of Surgery, Maastricht UMC, Maastricht, The Netherlands; 3 Trauma Research Unit, Department of Trauma Surgery, Erasmus MC, Rotterdam, The Netherlands

**Keywords:** xiphodynia, xiphoidectomy, sternum, thoracic surgery

## Abstract

**Introduction** Treatment options for xiphodynia are injections with local corticosteroid injections or surgical resection of the xiphoid process. Currently, there is no consensus as to which treatment is the most optimal.

**Objectives** The aim of this case series was to compare the safety and efficacy of conservative and surgical treatment for patients with xiphodynia.

**Patients and Methods** A retrospective case series was performed. All patients presenting with xiphodynia between 2016 and 2021 were eligible. Demographic data and treatment regimes, including preoperative work-up and surgical technique, were extracted from the electronic patient files. In addition, all patients received a follow-up phone call with a questionnaire. Patient satisfaction was measured using the Numeric Rating Scale.

**Results** A total of five patients, suffering from xiphodynia for up to 10 years, completed the follow-up questionnaire (median patient age, 57 years; range 51–68 years). Three of these patients initially received conservative treatment with local injections with corticosteroids for at least 6 months. One patient was satisfied with the results and did not opt for surgical treatment. Eventually, four patients were treated surgically by removing the xiphoid process. No postoperative complications were recorded and 100% of the patients who underwent a xiphoidectomy were free of symptoms and satisfied with the results.

**Conclusion** Symptoms related to xiphodynia can be relieved using conservative or surgical treatment, where the latter seems to be a safe and effective solution.

Xiphodynia is an uncommon thoracic wall disease, characterized by local tenderness over the xiphoid process. Patients can report symptoms such as thoracic or abdominal wall pain, throat tightness, nausea, and referred pain to arms, shoulders, back, or neck. 1 2 3 Symptoms can often be provoked by manual compression of the sternum and the xiphoid process, which also serves as a confirmation of the diagnosis. Additionally, a chest X-ray, upper abdominal ultrasound, or conventional computed tomography (CT) scan may show a displaced or protruded xiphoid process. 4 5 However, xiphodynia should be considered a diagnosis per exclusionem, as symptoms can mimic life-threatening diagnoses such as myocardial infarction. 6

The exact incidence or prevalence of xiphodynia is yet to be established. In 1955, Lipkin et al found the thoracic wall disease present in 2% of the general hospital population; however, recent numbers are not available. 1 The precise etiology of xiphodynia is currently unknown. Multiple factors have been postulated to be of influence, including excessive change in weight, previous abdominal or thoracal surgery, inflammation due to mechanical injury, repetitive minor trauma, or an anatomical anomaly. 3 4 7 8 Xiphodynia can be treated conservatively with physiotherapy, oral analgesics, or local corticosteroid injections. 1 3 4 5 8 9 10 Surgical treatment comprises removal of the xiphoid process (xiphoidectomy) and seems to have promising results. 3 4 5 9 Previous studies indicate that a xiphoidectomy is an outstanding treatment option, but that more evidence supporting this statement is required. 4 5 8 10 Also, a comparison between conservative and surgical treatment is lacking. Therefore, the aim of this case series was to compare the safety and efficacy of conservative and surgical treatment for patients with xiphodynia.

## Materials and Methods

### Study Design

A retrospective case series was performed in a local teaching hospital (Alrijne Hospital, Leiderdorp, the Netherlands). The study protocol was reviewed by the hospital's local medical ethical committee and approved under the number NWMO 21–19.

### Study Population

All patients presenting with sternal pain at the hospital's surgical outpatient clinic between 2016 and 2021 were eligible. A search for the International Classification of Diseases, 10th revision, code S23.4, “Sprain and strain of ribs and sternum,” was performed in the electronic patient files of patients visiting the outpatient clinic. Patients were included if pain symptoms could be provoked by direct palpation of the xiphoid process. Also, other underlying pathologies had to be excluded (e.g., myocardial infarction, pulmonary embolism, aortic aneurysm, pericarditis, or reflux disease). 3 6 10 11 As evidence comparing surgical to conservative treatment for xiphodynia is sparse, patients were counseled on both treatment options. The decision regarding preferred treatment was made together with an experienced clinician. Aside from missing data, no other exclusion criteria were drafted. Informed consent was obtained from all patients.

### Data Collection

Patient demographics were retrospectively extracted from electronic patient files. In addition, all patients were contacted via phone to conduct a follow-up questionnaire. All patients gave informed consent, agreeing to the scientific and anonymous use of relevant clinical data. Patient satisfaction with treatment was measured using the Numeric Rating Scale ranging from 1 to 10, with 1 being very dissatisfied and 10 being the most content imaginable.

### Conservative Treatment

Three patients received a periosteal methylprednisolone acetate–lidocaine (40 mg/10 mg) infusion, injected on both sides of the xiphoid process. A total amount of 1 mL was injected. Conservative treatment was tried for at least 6 months. When pain relief could not be optimally established, surgical treatment was offered to the patient.

### Surgical Treatment

A xiphoidectomy was performed under general anesthesia, with the patient in supine position. A 3- to 4-cm longitudinal midline incision was made above the xiphoid process. After opening the abdominal fascia, the xiphoid process was exposed and displaced or protruded parts of the xiphoid process were excised. Bone-nibbling forceps were used for correcting any irregularities. The abdominal fascia was closed with a PDS loop and the subcutis and skin were closed using intracutaneous sutures.

## Results

### Patient Characteristics

Between 2016 and 2021, a total of five patients presenting with xiphodynia met all inclusion criteria and consented to participation. Patient characteristics are listed in Table 1 . Three male and two female patients were included with a median age of 57 years (range, 51–68 years) and a mean body mass index of 30.0 kg/m ^2^ . One patient had a previous lower abdominal wall correction, which was closed primarily. Suggested reasons for the experienced xiphodynia were blunt trauma of the chest wall during a cycle accident ( *n* = 1) and a significant weight gain secondary to a change in therapeutic regimen for Crohn's disease ( *n* = 1). One case initially suffered from bacterial gastritis and heavy reflux, which did not disappear after starting sufficient treatment with medication.

**Table 1:** Overview of patient characteristics

Patient ID	Age (y)	Sex (male/female)	Body mass index (kg/m ^*2*^ )	Relevant medical history	Duration of symptoms (y)	Main symptom	Possible reason for xiphodynia	Additional imaging
1	61	Male	–	Hypertension	2	Pain when sitting and lying in prone position	None	CT scan
2	57	Male	25	None	3	Pain when lying in prone position	Trauma	–
3	51	Female	36	Morbus Crohn, preventive mastectomy	2	Pain when lying in prone position	Weight gain	Ultrasound
4	56	Male	36	Hypertension	10	Pain when sitting, lying, or bending over	None	Ultrasound, X-thorax
5	68	Female	23	Abdominal wall correction, reflux, cicatricial hernia correction, sigmoid resection, *Helicobacter pylori* infection	0.5	Pain from direct pressure on the chest wall	Heavy reflux, gastritis	Ultrasound, X-thorax, CT scan

Abbreviation: CT, computed tomography.

Upon performance of a diagnostic ultrasound of the sternum ( *n* = 3), a ventrally angulated and sensitive xiphoid process was found. Similar findings were found on the CT images ( *n* = 2) and conventional imaging ( *n* = 2) performed prior to treatment. In current clinical practice, no additional imaging was performed after treatment since this is not part of standard care.

### Treatment Outcome

The mean follow-up time after treatment was 2 years and 4 months ( Table 2 ). Three of the five participants initially received analgetic injections for at least 6 months. Positive effects were reported by all three in the first days after the first injection. One patient remained satisfied with the results, as daily activities could be performed without complaints. The two other patients indicated to have recurrence of complaints after two trials of infiltrations and opted for surgical treatment.

**Table 2:** Treatment outcome following conservative or surgical treatment of xiphodynia

Patient ID	Initial treatment	Number of injections	Definite treatment	Complications	Satisfaction with final treatment (1–10)	Free of symptoms
1	Conservative	3	Conservative	–	7	No
2	–	–	Xiphoidectomy	No	9	Yes
3	–	–	Xiphoidectomy	No	10	Yes
4	Conservative	2	Xiphoidectomy	No	10	Yes
*5*	Conservative	2	Xiphoidectomy	No	9	Yes

A total of four patients underwent a xiphoidectomy. The mean operating time was 19 minutes (range 12–27 minutes). None of the patients experienced pre- or postoperative complications. After a mean follow-up of 28 months after surgery, 100% of patients were free of symptoms and satisfied with the treatment.

## Discussion

Xiphodynia is a rare diagnosis, characterized by pain of the xiphoid process. The current case series of five patients who were treated for xiphodynia shows that an improvement of symptoms could be achieved using either conservative treatment, surgical intervention, or both. However, variation in treatment outcome was found for patients receiving conservative treatment, ranging from no effect to being relatively pain-free. In contrast, 100% of the patients who underwent a xiphoidectomy showed a significant improvement of complaints and were very satisfied with treatment.

In essence, permanent removal of the painful xiphoid seems an obvious and safe long-term solution to xiphodynia. Previous studies showed that a xiphoidectomy is an effective surgical procedure with improvement of symptoms in 89 to 100% of patients. 4 5 8 10 Findings of the current study further support this outcome, as all patients were satisfied with the result and had complete resolution of symptoms and no pre- or postoperative complications were reported. Yet, in this case series, one patient did not require surgical treatment and experienced less symptoms after an anesthetic corticosteroid injection. This suggests that analgesic infiltrations offer a sufficient treatment option and should be considered before opting for surgery.

**Fig. 1:**
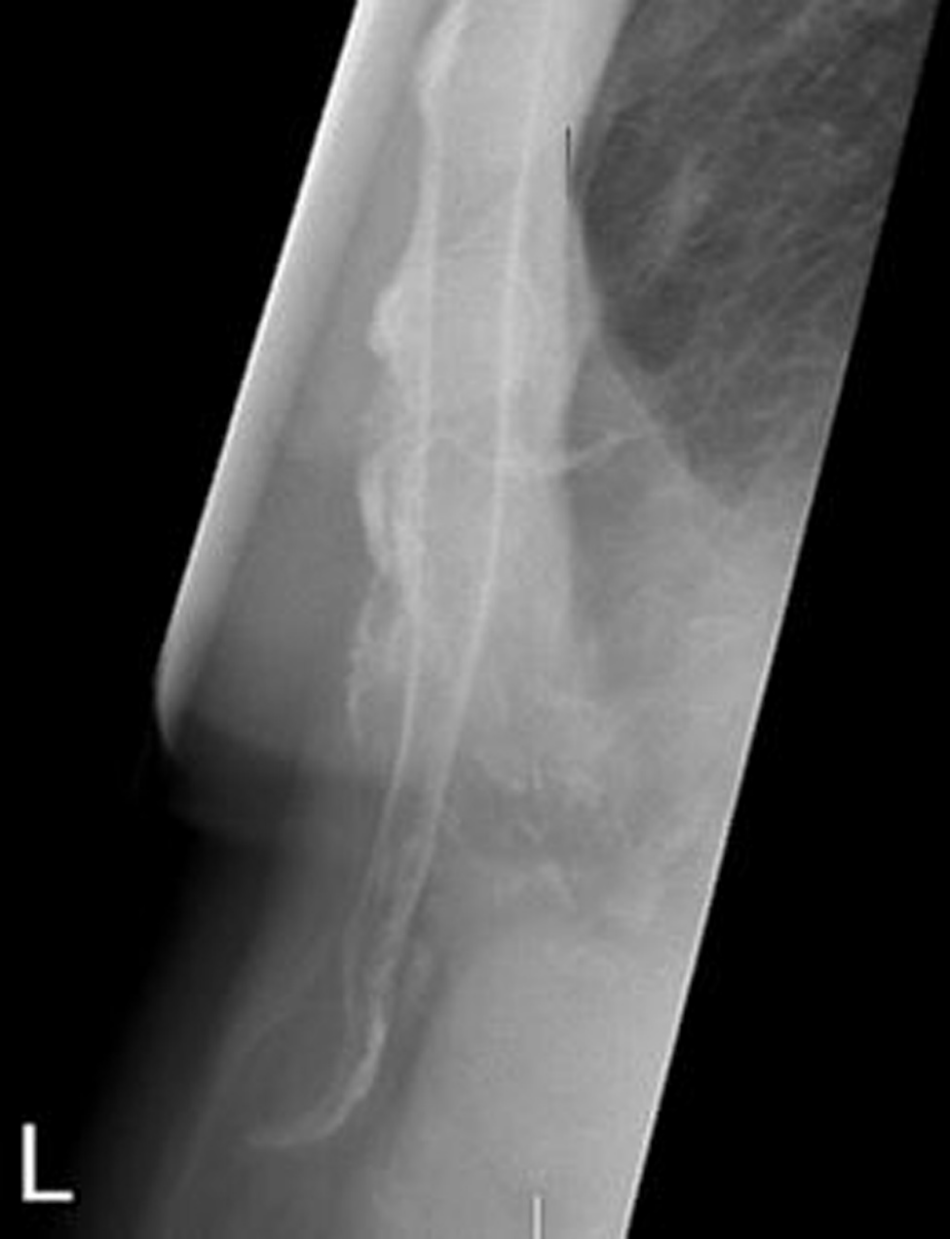
Example of a prominently displaced xiphoid process on a sagittal image of the chest wall.

A xiphoidectomy should only be performed when it is inevitable that the xiphoid process is responsible for the complaints. As mentioned before, potentially life-threatening thoracoabdominal diagnoses should be carefully excluded before considering xiphodynia. Even though tenderness over the xiphoid upon palpation and an angulating xiphoid on cross-sectional imaging are important indicators for the diagnosis Fig. 1 , obtaining absolute certainty remains challenging. To strengthen the diagnosis, a simple and standardized trial of bilateral infiltrations with anesthetic corticosteroids can be of diagnostic value. At the same time, this trial prevents surgical treatment when sufficient pain relief can be achieved conservatively, as is the case with other pain syndromes such as ACNES (anterior cutaneous nerve entrapment syndrome). While other conservative therapies have previously been proposed, such as oral analgesics and physiotherapy, future studies should evaluate whether improvement of complaints after local injections not only can serve as diagnostic value, but also can provide for substantial pain relief and thereby prevent surgical treatment. 4 7 9

Besides anatomic anomalies, factors predisposing for xiphodynia could include chest trauma, significant weight gain, or repeated mechanical injury. 8 12 Also, middle-age seems to be a predisposing factor for symptomatic xiphodynia. 5 8 10 These factors appear to be in concordance with current findings, since the median age was 57 years and mechanisms reported prior to start of complaint included trauma to the chest wall and excessive weight gain. Moreover, three of five patients were male. Xiphodynia can therefore be added to the differential diagnosis in middle-aged, male patients experiencing chest pain after a period of trauma in the sternal area or new-onset adiposity. However, studies with a bigger patient population should be performed to confirm these predisposing factors.

The retrospective nature of this study comes with several limitations, most importantly being the absence of a standardized treatment protocol. Therefore, not all included patients received standardized analgetic injections prior to surgical treatment. Numerous reasons could have been of influence, such as certainty about the diagnosis, patient treatment preferences, and level of symptoms. Also, as is to be expected from a single-center study covering a rare and underrecognized disease, the sample size is relatively small. Due to this limited number of participants, no other conservative treatment options were included. Nevertheless, studies comparing conservative versus operative treatment for xiphodynia are scarce and therefore provide the only available evidence. Future research should focus on the effectiveness of a standardized conservative treatment, compared with a xiphoidectomy.

## Conclusion

Symptoms related to xiphodynia can be relieved using conservative or surgical treatment, where the latter seems to be a safe and effective solution.
